# R-CHOP with dose-attenuated radiation therapy could induce good prognosis in gastric diffuse large B cell lymphoma

**DOI:** 10.1186/2162-3619-1-30

**Published:** 2012-09-24

**Authors:** Yuko Mishima, Yasuhito Terui, Masahiro Yokoyama, Noriko Nishimura, Sakura Sakajiri, Kyoko Ueda, Yasutoshi Kuboki, Kenji Nakano, Kazuhito Suzuki, Eriko Nara, Naoko Tsuyama, Kengo Takeuchi, Masahiko Oguchi, Kiyohiko Hatake

**Affiliations:** 1 Division of Hematology, Cancer Institute Hospital, Japanese Foundation for Cancer Research, 3-8-31 Ariake, Koto-ku, Tokyo, 135-8550, Japan; 2 Division of Pathology, Cancer Institute Hospital, Japanese Foundation for Cancer Research, 3-8-31 Ariake, Koto-ku, Tokyo, 135-8550, Japan; 3 Radiation Oncology Department, Cancer Institute Hospital, Japanese Foundation for Cancer Research, 3-8-31 Ariake, Koto-ku, Tokyo, 135-8550, Japan

**Keywords:** Gastric DLBCL, Radiation, R-CHOP

## Abstract

**Background:**

The treatment strategy for gastric diffuse large cell lymphoma (DLBCL) has not been standardized in such as to the cycles of chemotherapy, dose of radiation, or necessity for the surgery. Although the results of CHOP or R-CHOP treatments have demonstrated the good prognosis, the treatments have been controversial in many cases.

**Methods:**

We retrospectively analyzed 40 gastric DLBCL patients receiving chemotherapy with or without radiation in our institute. Those in stages II-IV were treated with six cycles of R-CHOP without radiation; for those in stage I, we administered three cycles of R-CHOP with radiation.

**Results:**

The three-year overall survival (OS) and progression-free survival (PFS) rates were 95.2 and 91.8%, respectively. Those in stage I obtained 100% of OS. The radiation dose prescribed was 30.6 Gy for CR cases and 39.6 to 40 Gy for PR after chemotherapy. Although survival rates tended to correlate with staging groups or age-adjusted IPI classifications, multivariate statistical analysis did not show clear differences. All 14 patients with initial bleeding were successfully managed without surgery during treatment.

**Conclusion:**

R-CHOP therapy was very effective for gastric DLBCL. It may be not necessary to use more than 30.6 Gy of radiotherapy in the highly chemo-sensitive cases. Less toxic treatments should be made available to gastric DLBCL patients.

## Background

Primary gastric diffuse large B cell lymphoma (DLBCL) is a relatively common disease in gastric lymphoma as well as gastric marginal zone B cell lymphoma (MALT). In many previous reports, the prognosis for gastric lymphoma was considered good; however, these reports involved MALT, indolent lymphoma, and DLBCL cases [1,2]. Treatments were varied and included surgical resection, radiotherapy, antibiotics against helicobacter pyroli, and chemotherapy [3]. Reports focusing only on primary gastric DLBCL are very few, and the treatment strategy has not been stabilized and individualized across institutions. Recently, it has been known that rituximab combined CHOP (R-CHOP) has been shown to be very effective for DLBCL, remarkably improving overall survival and progression free-survival [4]. For localized DLBCL, radiation following limited cycles of R-CHOP led to good prognosis [5-7]. In the Southwest Oncology Group Study (SWOG) 0014 study, RCHOP with radiotherapy for early-stage gastric DLBCL demonstrated a good prognosis [8]. Ferrucci summarized in his review using three to four cycles of R-CHOP followed by involved field radiotherapy in early-stage of DLBCL and using six to eight cycles of R-CHOP alone in advanced-stage patients [9].

In our study, we retrospectively analyzed our patients with primary gastric DLBCL without resection and examined the possibility of using less-toxic treatment.

## Patients and methods

### Patient eligibility and diagnosis

We retrospectively analyzed 40 patients diagnosed with gastric DLBCL in our institute between November 2003 and October 2008. The tissue specimens were obtained from the biopsies of gastric tumors using upper endoscopy and were diagnosed using immunohistochemical staining and flowcytometry by expert hematological pathologists. Disease stages were based on the Lugano classification [10], using computed tomography (CT), ultra-sound examination (US), positron emission tomography with or without CT (PET/CT), and bone marrow aspiration and biopsy. In the cases with other invasions in nodal or extra nodal sites, if the cases had predominant lesions in stomach, we diagnosed as primary gastric lymphoma [11]. Cases with gastric mass exceeding more 7 cm were classified as bulky disease.

### Treatment and evaluation

Treatment strategy was classified dependent on stage. Patients with stage I but not bulky disease were treated with three cycles of R-CHOP; eight doses of rituximab (375 mg/m^2^ weekly) and three cycles of CHOP (Cyclophosphamide 750 mg/m^2^, day 1; vincristine 1.4 mg/m^2^, day 1; doxorubicin 50 mg/m^2^, day 1; and Prednisolone 60 mg/m^2^, days 1-5; tri-weekly) followed by involved field radiotherapy. Patients with stage II-IV were treated with six cycles of R-CHOP (eight cycles of weekly rituximab and six cycles of CHOP) without radiotherapy. Patients with bulky disease in either stage were treated with six cycles of R-CHOP followed by radiotherapy. CHOP doses were decreased to 80 percent in patients older than 75 years old, and in the patients experienced with grade 4-hematological toxicity, and/or grade 3 or more grade of non-hematological toxicity. G-CSF supports were administered to patients older than 70 and to patients who had FN in previous cycles. Basically, all patients received an endoscopy after the first cycle of chemotherapy and after completing the treatment. PET scans were performed after 3 cycles of chemotherapy in the limited stage. Response to total treatment was evaluated using CT scans four weeks after and using PET/CT scans and endoscopy six weeks after the last treatments.

### Radiation therapy

**Figure 1:**
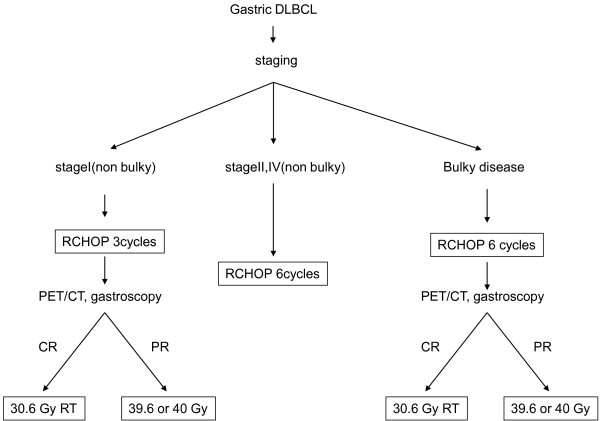
The schema of treatment of gastric DLBCL in our study.

Three-dimensional conformal radiation planning (Eclipse, Varian Medical Systems, Palo Alto, CA.) based on CT with/without respiratory gating was required for all patients for whom radiation therapy was indicated. The clinical target volume of radiotherapy encompassed the entire stomach and involved the perigastric or abdominal lymph node regions if nodal lesions were present. We also added an appropriate margin to the clinical target volume taking into account respiratory gastric movements. The prescribed dose of radiotherapy was 30.6 Gy in 17 fractionations over 3.5 weeks for CR cases and 39.6 Gy in 23 fractionations over 4.5 weeks or 40 Gy in PR case (Figure 1). Radiation therapy was delivered with a 10 or 15 MV photon beam linear acceralator (CL2100, Varian Medical Systems, Palo Alto, CA.).

### Ethics of treatment design

Our treatment design was based on NCCN guidelines for diffuse large lymphoma [12]. Radiation doses were selected according to NCCN guidelines in which recommended dose were 30 Gy in CR cases and 40 Gy in PR cases. Decisions on initial treatment for all patients with gastric bleeding or having risk of perforation were discussed by a lymphoma cancer board comparing hematologists, surgeons, radiologists, and radiation oncologists.

### Follow-up and statistical analysis

All patients were followed by medical oncologists and by radiation oncologists every three months for two years, then every six months for the next three years, with repeated CT scans and/or endoscopy.

Statistical analyses of progression-free survival (PFS) and overall survival (OS) were calculated using Kaplan-Meier estimators. Categories were compared by means of a log-rank test. As risk factors, we included age-adjusted (aa) IPI, disease stage, GC vs non-GC subtype, hemoglobin level (Hb < 12.0 g/dl), stage, bleeding, CRP level, be-ta 2 microglobulin, and IL-2 as risk factors. Taking stage-risk in IPI score into consideration, we estimated Lugano-stage IV as high risk and I, II as low risk. Using Cox proportional hazards regression analysis, univariate and multivariate analysis were performed on these risk factors.

## Results

### Patient characteristics and prognostic factors

Patient characteristics are shown in Table 1. In total, 40 patients were analyzed in this study. The median age was 65.5 years (30-79 years old), and 21 patients (52.5%) were male. For aa IPI, low was 23, low intermediate was 8, high intermediate was 3, and high was 6 cases. The patients with Stage IV involved extensive lesions such as lung, liver, and mediastitial lymph nods. GC and non-GC types were 23 (75%) and 10 (25%), respectively. For the level of hemoglobin (Hb), lower than 12 g/dl was 11, and bleeding or deep ulcer cases at diagnosis were fourteen. Thirty-five of 40 patients completed the planned treatment.

**Table 1:** Patient characteristics

**Patient characteristics**	**No. of patients**
median age	65.5 (30-79 years)
M/F	21/19
aa-IPI	
L	23 (57.5%)
LI	8 (20%)
HI	3 (7.5%)
H	6 (15%)
stage	
I (Ix)	15 (2) (37.5%)
II II1	8 (20.0%)
II2	3 (7.5%)
IIE	2 (5.0%)
IV	12 (30%) *
GC/non-GC	
GC	23 (57.5%)
non-GC	10 (25.0%)
unclassified	7
Hb	
<12	12 (30%)
>12	28 (70%)
treatment	
3 cycles R-CHOP + RT (30 Gy)	11 (27.5%)
6 cycles R-CHOP	21 (52.5%)
6 cycles R-CHOP + RT (30 Gy)	3 (7.5%)
drop out	5 (12.5%)

*involved site; liver (4) lung, pleural effusion (5) mediastitial LNs (3).

Abbrebiations; *GC*; germinal center type, *RT*; radiotherapy.

### Treatment feasibility

Fifteen patients were stage I (27.5%), including two with bulky disease, and 11 patients received three cycles of R-CHOP (rituximab was 8 cycles, weekly), followed by radiation. The two with bulky disease received 6 cycles of R-CHOP and radiation. Two patients received only 3 cycles of RCHOP alone because of their complications and concomitant disorders.

Twenty-one patients (72.5%) were stage II and IV (stage II was 13, IV was 12) and all of them were treated with 6 cycles of RCHOP (rituximab was 8 cycles, weekly). One case of stage II with bulky disease received 6 cycles of R-CHOP with consolidative radiation therapy.

Of the patients receiving radiotherapy, nine patients with CR after chemotherapy received 30.6 Gy, and five patients with PR received 39.6 Gy or 40 Gy.

In the fourteen patients with initial presentation of bleeding from ulcerative lymphoma lesions, there were none with repeated gastric bleeding or perforation after chemotherapy.

### Initial treatment response and survival

**Figure 2:**
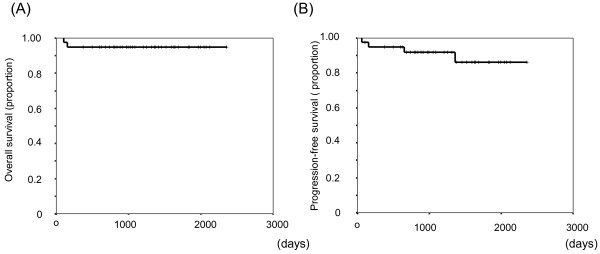
Kaplan-Meier curve of overall survival (A) and progression free survival (B) for all patients.

**Figure 3:**
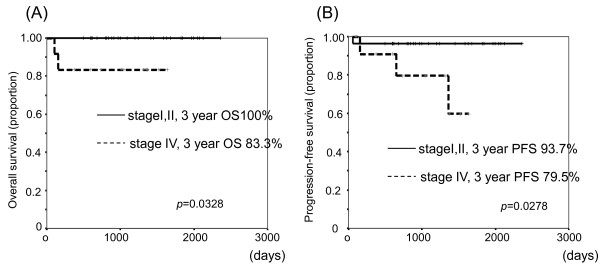
Overall survival (A) and progression free survival (B) in classified with stage I,II and IV.

**Figure 4:**
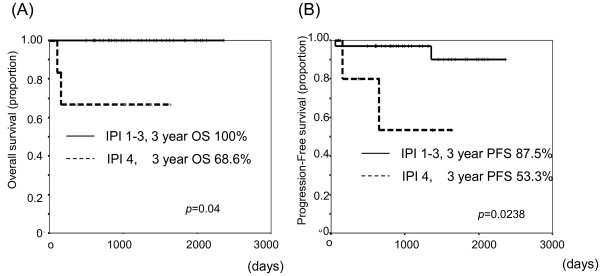
Overall survival (A) and progression free survival (B) in classified with aa-IPI 1-3 and 4.

The average duration of observation duration was 42.5 months. The complete remission (CR) rate overall was 87.5% (35/40), and in only one patient did disease progress (PD) during R-CHOP treatment. One patient relapsed after obtaining CR. The relapse sites were para-aortic and peritoneal lymph nodes. There was no gastric invasion. Three-year OS and PFS rates of the study population were 95.2% and 91.8%, respectively (Figure 2). According to the prognostic factors, three-year-OS was 100% and 83.3% in stages I-II and IV, respectively. And that of PFS was 100%, 92.3%, and 79.5% in stages I, II, and IV, respectively (Figure 3). Otherwise, three-year OS dependent on aa-IPI was 100% and 68.6% in 1-3 and 4, respectively. That of PFS was 87.5% and 53.3% in aa-IPI 1-3 and 4, respectively (Figure 4). According to the treatment regimen, three-year-OS and PFS was 100% and 100% in all three cycles R-CHOP with radiation, 91.3% and 87.0% in six cycles R-CHOP, 100% and 100% in six cycles R-CHOP with radiation, respectively.

Univariate statistical analysis of IL-2, be-ta 2 microglobulin, CRP, GC or non-GC subtype did not show significant differences in survival. However, there were marginally significant differences between stage I-II and IV (OS; *p* = 0.0328, PFS; *p* = 0.0273) (Figure 3) and between aa-IPI 1-3 and 4 (OS; *p* = 0.004, PFS; *p* = 0.0238) (Figure 4), These survival differences were not significant in multivariate analysis (data not shown).

### Treatment toxicity

Adverse events greater than grade3 are shown in Table 2. The main adverse events were grade 4 neutropenia 40.0% and febrile neutropenia 17.5%. Those who dropped out from these regimens did so because of psychosis, colitis, myelosuppression, intestitial pneumonia, and liver damage, one case for each of these. Both cases with psychosis and colitis were 79 years old, both were stages IV, and they had severe performance status at the diagnosis. There was no CNS invasion or intestinal invasion. The case with grade3 liver toxicity was induced in the first cycle of R-CHOP and had no liver invasion. The case with severe myelosupression had no bone marrow invasion but was stage IV. One patient with stage IIE had interstitial pneumonia due to Pneumocystis Jiroveci after 5 cycles of R-CHOP. No acute and delayed radiation-related toxicities, such as chronic gastric ulcer, liver dysfunction, and renal insufficiency, graded 3 or higher, were documented during the observation period.

**Table 2:** Treatment result and adverse events

**Treatment result**	**No. of patients (total number of patients = 40)**
complete treatment	35
CR	35 (87.5%)
PD	1
relapse	1
death	2
adverse events	
Hematologic toxicity (grade4)	
neutropenia	12(40%)
Non-Hematological toxicity (grade3 and 4)	
febrile neutropenia	7 (17.5%)
liver toxicity (GOT/GPT elevation)	1
colitis	1
psycosis	1
SIADH	1
interstitial pneumonia	2

Abbrebiations; *CR*; complete remission, *PD*; progressive disease.

## Discussion

R-CHOP has been recognized as standard therapy of DLBCL. In Wohrer’s report, R-CHOP for the early stage of gastric DLBCL resulted in 87% CR, with 12 of 15 patients alive at 15 months after chemotherapy [13]. Although our study was small sample size, all the stage I patients obtained CR, with both OS and PFS at 100%, and also stage II-IV patients obtained long survival. We should consider cases with bleeding and/or potential bleeding from deep ulcerative lesions, always paying attention to the possibility of repeated bleeding or perforation of stomach after chemotherapy. Although such cases were included in this study, we were able to complete R-CHOP safely.

The dose of radiotherapy should be discussed. In the SWOG0014 study, the dose of involved field radiation was 40-46 Gy [6]. A Japanese multi-center phase II trial reported using 40.5 Gy of radiation [14]. In another phase II study, four cycles of CHOP with IFRT of 40 Gy was adjusted to early stage of primary gastric DLBCL [15]. The IELSG4 study compared chemotherapy only without rituximab treatment versus chemotherapy with involved field radiotherapy. In this study, cases were limited to stage I to stageIIE, and they evaluated after four cycles of CHOP-like regimens. Then if the cases obtained CR, they were randomized to an additional two cycles of chemotherapy and 30 Gy involved field RT. There were no significant differences in OS between the two arms, though DFS was significantly better than radiotherapy groups, and four cases (18%) relapsed in the chemotherapy-alone group [16]. In our study, we planned the dose of radiation as 30.6 Gy in CR cases and 39.6-40 Gy in PR cases based on NCCN guidelines for DLBCL [12] and there were no relapses. This consensus was based on expert opinion that the volume and dose of radiation therapy should be as minimimal as possible because the gastric DLBCL is surrounded with at-risk organs such as liver, kidneys and heart. Radiation-induced acute toxicities such as nausea and appetite loss and delayed toxicities such as gastric ulcer and renal or liver dysfunction could be reduced by lower dose of radiation. The risk of secondary malignancy should be evaluated after 10 years following this treatment. It is a very important point of treatment for gastric DLBCL, although it is difficult to conduct a randomized study specifically focusing on the doses of radiation therapy, mainly because of patient accrual.

Furthermore, we administered eight doses of rituximab in the treatment, compared to four doses in the SWOG0014 study [6]. To complete infusion of eight cycles of rituximab during chemotherapy, we decided on weekly administration of rituximab. The eight doses of rituximab might have contributed to our good results.

In this our present report, most patients dropping chemotherapy were stage IV and high score of aa-IPI, and their case were complicated by severe general condition at diagnosis. All patients who completed R-CHOP with or without radiotherapy were able to obtain long PFS and OS. We therefore recommend R-CHOP therapy as a promising treatment for gastric lymphoma, carefully selecting the dose of radiotherapy dependent on chemo-sensitivity.

## Competing interests

The authors declare that they have no competing interests.

## Author’s contributions

MY, TY, YY, SY, NN, KU, YK, KN, KS, EN and HK collected and reviewed data to compile into the paper. TN and TK reviewed and advised pathologic data. OM reviewed and advised radiologic data. All the authors reviewed and approved the final version of this report.
